# Correction: Dual functions of a small regulatory subunit in the mitochondrial calcium uniporter complex

**DOI:** 10.7554/eLife.26228

**Published:** 2017-03-02

**Authors:** Ming-Feng Tsai, Charles B Phillips, Matthew Ranaghan, Chen-Wei Tsai, Yujiao Wu, Carole Willliams, Christopher Miller

Tsai M-F, Phillips CB, Ranaghan M, Tsai C-W, Wu Y, Williams C, Miller C. 2016. Dual functions of a small regulatory subunit in the mitochondrial calcium uniporter complex. *eLife* **5**:e15545. doi: 10.7554/eLife.15545.

Published 21, April 2016

The protein sequence of EMRE from *D. melanogaster*, sequence #13 in Supplementary file 1, was incorrect due to a mistaken copy-and-paste while preparing the manuscript. The correct *D. melanogaster* sequence is now entered in the corrected Supplementary file 1. We are grateful to J. Agapite, a curator of FlyBase, who noticed the error and reported it to the senior author.

This error did not affect any results or conclusions of the paper.

Incorrect sequence:

MTSKTVFQNAFKTFLDFAINSLPSTQGGLNITATAPGGVGQRPFTNKAGVLKLIFVSASSLYIGGLIAHKGASYLEENEIFVPTDEDDDD

Corrected sequence:

MIVPRLALPISLALQRVSRRVAEHPHNLRILQRHMSSVYFRSGAIKPKPEEMPFGLLAIFCAVIPGLFVGATISKNVANFLEENDLFVPADDDDDED

The article has been corrected accordingly.

## Additional files

Supplementary file 1. Supplementary experimental procedure.

