# Supplementary material for: Dual functions of a small regulatory subunit in the mitochondrial calcium uniporter complex
Source: eLife. 2016 Apr 21;5:e15545. doi: 10.7554/eLife.15545 (PMC4892889; doi:10.7554/eLife.15545)
Supplement: Supplementary file 1. — DOI: http://dx.doi.org/10.7554/eLife.15545.017 [file elife-15545-supp1.docx]

**Supplementary File 1**

*Sequences of primers used for qPCR*

β-actin forward: ggcatgggtcagaaggatt

β-actin reverse: ccacacgcagctcattgta

MICU1 forward: cgatccataacacccaatga

MICU1 reverse: tctgtctttccatcaaagcg

*Amino acid sequences of mutant proteins used in this study*

1. Human MCU

MAAAAGRSLLLLLSSRGGGGGGAGGCGALTAGCFPGLGVSRHRQQQHHRTVHQRIASWQNLGAVYCSTVVPSDDVTVVYQNGLPVISVRLPSRRERCQFTLKPISDSVGVFLRQLQEEDRGIDRVAIYSPDGVRVAASTGIDLLLLDDFKLVINDLTYHVRPPKRDLLSHENAATLNDVKTLVQQLYTTLCIEQHQLNKERELIERLEDLKEQLAPLEKVRIEISRKAEKRTTLVLWGGLAYMATQFGILARLTWWEYSWDIMEPVTYFITYGSAMAMYAYFVMTRQEYVYPEARDRQYLLFFHKGAKKSRFDLEKYNQLKDAIAQAEMDLKRLRDPLQVHLPLRQIGEKD

1. Human EMRE

MASGAARWLVLAPVRSGALRSGPSLRKDGDVSAAWSGSGRSLVPSRSVIVTRSGAILPKPVKMSFGLLRVFSIVIPFLYVGTLISKNFAALLEEHDIFVPEDDDDDD

1. Human MICU1

MFRLNSLSALAELAVGSRWYHGGSQPIQIRRRLMMVAFLGASAVTASTGLLWKRAHAESPPCVDNLKSDIGDKGKNKDEGDVCNHEKKTADLAPHPEEKKKKRSGFRDRKVMEYENRIRAYSTPDKIFRYFATLKVISEPGEAEVFMTPEDFVRSITPNEKQPEHLGLDQYIIKRFDGKKISQEREKFADEGSIFYTLGECGLISFSDYIFLTTVLSTPQRNFEIAFKMFDLNGDGEVDMEEFEQVQSIIRSQTSMGMRHRDRPTTGNTLKSGLCSALTTYFFGADLKGKLTIKNFLEFQRKLQHDVLKLEFERHDPVDGRITERQFGGMLLAYSGVQSKKLTAMQRQLKKHFKEGKGLTFQEVENFFTFLKNINDVDTALSFYHMAGASLDKVTMQQVARTVAKVELSDHVCDVVFALFDCDGNGELSNKEFVSIMKQRLMRGLEKPKDMGFTRLMQAMWKCAQETAWDFALPKQ

1. Human MICU2

MAAAAGSCARVAAWGGKLRRGLAVSRQAVRSPGPLAAAVAGAALAGAGAAWHHSRVSVAARDGSFTVSAQKNVEHGIIYIGKPSLRKQRFMQFSSLEHEGEYYMTPRDFLFSVMFEQMERKTSVKKLTKKDIEDTLSGIQTAGCGSTFFRDLGDKGLISYTEYLFLLTILTKPHSGFHVAFKMLDTDGNEMIEKREFFKLQKIISKQDDLMTVKTNETGYQEAIVKEPEINTTLQMRFFGKRGQRKLHYKEFRRFMENLQTEIQEMEFLQFSKGLSFMRKEDFAEWLLFFTNTENKDIYWKNVREKLSAGESISLDEFKSFCHFTTHLEDFAIAMQMFSLAHRPVRLAEFKRAVKVATGQELSNNILDTVFKIFDLDGDECLSHEEFLGVLKNRMHRGLWVPQHQSIQEYWKCVKKESIKGVKEVWKQAGKGLF

1. MCU-EMRE fusion protein

MAAAAGRSLLLLLSSRCGGGGGAGGCGALTAGCFPGLGVSRHRQQQHHRTVHQRIASWQNLGAVYCSTVVPSDDVTVVYQNLEGEWQNLGAVYCSTVVPSDDVTVVYQNGLPVISVRLPSRRERCQFTLKPISDSVGVFLRQLQEEDRGIDRVAIYSPDGVRVAASTGIDLLLLDDFKLVINDLTYHVRPPKRDLLSHENAATLNDVKTLVQQLYTTLCIEQHQLNKERELIERLEDLKEQLAPLEKVRIEISRKAEKRTTLVLWGGLAYMATQFGILARLTWWEYSWDIMEPVTYFITYGSAMAMYAYFVMTRQEYVYPEARDRQYLLFFHKGAKKSRFDLEKYNQLKDAIAQAEMDLKRLRDPLQVHLPLRQIGEKDVSAAWSGSGRSLVPSRSVIVTRSGAILPKPVKMSFGLLRVFSIVIPFLYVGTLISKNFAALLEEHDIFVPEDDDDDDTETSQVAPA

*Note:* the mitochondrial targeting sequence at the N-terminus of EMRE is removed to prevent digestion of the fusion protein by mitochondrial proteases.

1. ΔN-EMRE

MASGAARWLVLAPVRSGALRSGPSLRKDGDVSAAWPRGPDRPEGIEEPKPVKMSFGLLRVFSIVIPFLYVGTLISKNFAALLEEHDIFVPEDDDDDD

1. ΔC-EMRE

MASGAARWLVLAPVRSGALRSGPSLRKDGDVSAAWSGSGRSLVPSRSVIVTRSGAILPKPVKMSFGLLRVFSIVIPFLYVGTLISKNFAALLPRGPDRPEGIEE

1. WALP-EMRE

MASGAARWLVLAPVRSGALRSGPSLRKDGDVSAAWSGSGRSLVPSRSVIVTRSGAILPKPVKGWWLALALALALALALALALWWASKNFAALLEEHDIFVPEDDDDDD

1. *C. elegans* MCU

MARNGRCLVTPFVTAQRLANLRNTLWNRQQIAFSTTTASSSTSPIQESSSPLSIRFEYGLPLLDVPLPSRNEPCQFTMRPLSDTIGSLCEFLRQEDRGIDYVAVYGTNGVKLATCTSIEHLLQFGSFRLRLNDKFFDVTVPKTGTMPYDSDKLRQLDDLRATVASLHAALCVDEYKLSREKKLLLQLENAETLLAPLHDAKRKIEQECEAHTDRVMWAGFAAMGVQTGLFARLTWWEYSWDIMEPVTYFATYSTVCATFGYYLYTQQSFEYPSARERVYTKQFYRRAQKQNFDIEKYNRLVTEVDELRNQLKRMRDPLFQHLPVSYLSNLEAEK

1. *C. elegans* EMRE

MATSKTVFQNAFKTFLDFAINSLPSTQGGLNITATAPGGVGQRPFTNKAGVLKLIFVSAS SLYIGGLIAHKGASYLEENEIFVPTDEDDDD

1. MCU chimera with *C. elegans* MCU scaffold and hMCU TMH2

MARNGRCLVTPFVTAQRLANLRNTLWNRQQIAFSTTTASSSTSPIQESSSPLSIRFEYGLPLLDVPLPSRNEPCQFTMRPLSDTIGSLCEFLRQEDRGIDYVAVYGTNGVKLATCTSIEHLLQFGSFRLRLNDKFFDVTVPKTGTMPYDSDKLRQLDDLRATVASLHAALCVDEYKLSREKKLLLQLENAETLLAPLHDAKRKIEQECEAHTDRVMWAGFAAMGVQTGLFARLTWWEYSWDIMEPVTYFITYGSAMAMYAYFVMTQQSFEYPSARERVYTKQFYRRAQKQNFDIEKYNRLVTEVDELRNQLKRMRDPLFQHLPVSYLSNLEAEK

12. *D. Melanogaster* MCU

MSRNRAAMVSAFRLFLRPATTTTHRSLALRLAPGTTFALHLRPCHELQQHRSFASTAEDGETDKHKKPTTGDIYVEYVNGMPHMTVRLPSRNELCQFALKPISHNVGDLLAMLRAEDRGIDRAAVINKHGVRIASSCTIESLLDDSFSIQINNRTLDVNPPKRDKVTLESMDKVGDVRKVIAQLYEAFNVGEYQLEKSNQLAKELETLRYELEPLEEKKLELSKKAARRTNFMTWMGLGLMSVQFGILARLTWWEYSWDIMEPVTYFVTYGTTMAMYAYYCVTKREYMMEDVKNREFSLSLYRNAKKVQFDVEHYNELKRKSAELEYNLRRINDPLNMQLPSHLVRTQENTPPTLTEEKAERKYT

13. *D. Melanogaster* EMRE

MIVPRLALPISLALQRVSRRVAEHPHNLRILQRHMSSVYFRSGAIKPKPEEMPFGLLAIF

CAVIPGLFVGATISKNVANFLEENDLFVPADDDDDED

14. *A. thaliana* MCU

MWSVMGLVRRTAMSSTVNKASPVRSLLGGFRCLNVESKEEDEKKDMTVLEAKKLMRLVNVEDMKKKLIGMGDKEMVTYTTLIEASQGLGIARSLDEAHAFARVLDDAGVILIFRDKVYLHPHKVVDLIRKAVPLGLNPDDELIREEFDKMRSMKEEIDVLAHQQVRKILWGGLGYSVVQIGIFVRLTFWEFSWDVMEPITFFTTATGIIVGYAYFLMTSRDPTYQDFMKRLFLSRQRKLLKSHKFDAERFKELENKWKITSCSSSSCHANASIRNRVGVDLDLEDSLQSHHRD

15. *D. discoideum* MCU

MNSFVIRNGFGLVRTFNTRLFTTSTQNLEGELKTILGQAKVSKLQEKLKLDPRSKITFNDFKGIAKEVGIEEKEINSVSNALAQSGSIIYLPNSLNENLKTSVFTKPAHIYQSLEHILDIENKGVGLNKLIESKKSEINSLRQKIQPLEEKKQVIDRKAHRRATAIIWTGLGYCFAQAAILARLTWWDLSWDIIEPVSYFLTFGSVLIGYTYFTMTKTEFTYEALNHRLFSKRQDKLFKRNNFPKEDYENLVQAIDKKEKELKELELATKYDHTH

*Guide sequences used for gene knockout*

MCU-KO1: CAGGAGCGATCTACCTGCGG

MCU-KO2: TGAACTGACAGCGTTCACGC

EMRE-KO1: GGCTAGTATTGGCACCCGTC

EMRE-KO2: TACTAGCCAGCGAGCCGCTC

*shRNA sequences used for MICU1 knockdown*

sh1: CCGGGCAATGGCGAACTGAGCAATACTCGAGTATTGCTCAGTTCGCCATTGCTTTTTG

sh2: CCGGGCAGCTCAAGAAGCACTTCAACTCGAGTTGAAGTGCTTCTTGAGCTGCTTTTTG

sh3: CCGGGCAATGGCGAACTGAGCAATACTCGAGTATTGCTCAGTTCGCCATTGCTTTTTG

The pLKO1 lentiviral vectors for expressing these shRNAs were directly purchased from Sigma (sh1: TRCN0000299804, sh2: TRCN0000299805, sh3: TRCN0000053370)
